# The complete mitochondrial genome of a medicinal fungus, *Tolypocladium ophioglossoides*

**DOI:** 10.1080/23802359.2017.1285208

**Published:** 2017-02-16

**Authors:** Huang Fangliang, Li Yongquan, Chen Xinai

**Affiliations:** a College of Life Sciences, Zhejiang University, Hangzhou, China;; b College of Pharmaceutical Sciences, Zhejiang University, Hangzhou, China

**Keywords:** *Tolypocladium ophioglossoides*, mitochondrial genome, Hypocreales, Clavicipitaceae

## Abstract

As part of a genome sequencing project for *Tolypocladium ophioglossoides* (*T. ophioglossoides*), a complete mitochondrial (mt) genome was assembled as a single circular dsDNA of 35,159 bp. Conserved genes including the large and small rRNA subunits, 25 tRNA and 15 protein-coding genes, were identified. In addition, 4 non-conserved open reading frames (ncORFs) in the intergenic and intronic regions were also identified. Transcription analyses using RNA-seq validated the expression of most conserved genes and ncORFs. Seven introns (groups I and II) were found within conserved genes, accounting for 21% of the mitogenome. All structural genes are located on one strand and are apparently transcribed in one direction. The complete mt genomes of *T. ophioglossoides* would be useful for future investigation of genetics, evolution and clinical identification of *Tolypocladium* species.

*Tolypocladium ophioglossoides* is a species of fungus in the family Ophiocordycipitaceae. It is parasitic on fruit bodies of the truffle-like Elaphomyces, ectomycorrhizal fungi closely related to *Aspergillus* and *Penicillium* (Landvik et al. 1996). It is distributed throughout many parts of the Northern Hemisphere (Mains 1957). This fungus has been used as a traditional rare and precious medicine in China for centuries. In this study, we report the complete mitochondrial genome sequence of *T. ophioglossoides* to provide useful genetic information for the future research in biology and medicine.

*T. ophioglossoides* was provided by Key Laboratory of Microbial Biochemistry and Metabolism Engineering of Zhejiang Province. Genomic DNA was prepared using CTAB method as described previously (Sun et al. 2013). One short sequencing library (*ca*. 200 bp) and three mate-pair librarys (*ca*. 3, 8, 10 kb) were constructed and sequenced using ABI PGM platform. Genome assembly and annotation were performed by CLC Genomies Workbench and Mfannot tool with default settings. One contig representing the mitochondrial DNA was identified on the basis of extensive sequence similarity to known fungal mitochondrial genomes. The complete mitochondrial genome of *T. ophioglossoides* was deposited in GenBank database with the accession number KX455872.The complete mitochondrial genome sequence of *T. ophioglossoides* is 35,159 bp in length and AT content of 72.47%. It consists of 15 core mitochondrial protein-coding genes, 25 tRNA genes, 2 rRNA genes (small and large subunit rRNA) and 2 unique and separate open-reading frames (ORFs) encoding proteins without apparent homology to any known proteins. Protein-encoding genes include ATP-synthase subunits 6, 8, and 9 (atp6, atp8, and atp9), cytochrome oxidase subunits (cox1, cox2, and cox3), apocytochrome b (cob), and NADH dehydrogenase subunits (nad1, nad2, nad3, nad4, nad4L, nad5, and nad6) whose products involved in mitochondrial electron transfer, oxidative phosphorylation and mitochondrial protein synthesis. All 44 protein- and RNA-encoding genes are located on the same strands and are transcribed in one direction. No Tandem Repeat Finder (TRF) hits were found on *T. ophioglossoides* used by Dfam. The gene order of *T. ophioglossoides* was identical with that of the known representatives of *Metarhizium anisopliae* mitochondrial genomes.

The set of 25 tRNA genes code for all 19 standard amino acids. Seventeen tRNA genes are adjacent to rnl, 4 tRNA genes approach to rns, and 4 tRNA genes are located around three protein-coding genes (cob, cox1, and cox3). Seven introns invade three genes including rnl (two), cob (one), cox1 (four). All these introns belong to group I introns. Intronic proteins include ribosomal protein S3 and GIY endonucleases.

Phylogenetic analysis based on whole mitogenome sequences confirms *T. ophioglossoides* as a member of the fungal order Hypocreales. *T. ophioglossoides* is clustered together with *M. anisopliae* and *M. chamydosporia* within the family Clavicipitaceae according to our phylogenetic analysis (Figure 1), with consistent taxonomic status according to phylogenetic analysis of nuclear genes of Hypocreales (Zhang et al. 2016).

**Figure 1.**
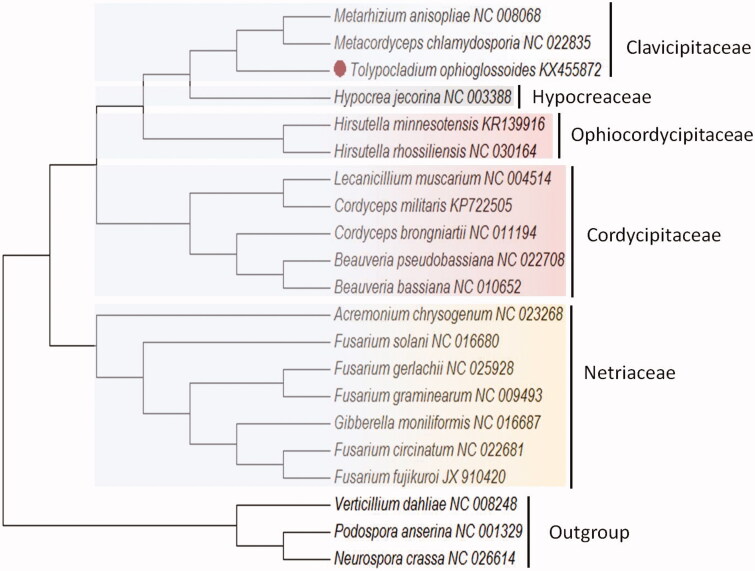
Phylogenetic analysis of *T. ophioglossoides* and mitochondrial genome of the related species. The ML-tree is based on 14 concatenated core mitochondrial proteins from 18 Hypocreales genomes. *Podospora anserine*, *Neurospora crassa*, and *Verticillium dahliae* were used as the outgroup. The evolutionary history was inferred using the neighbour-joining method. Evolutionary analyses were conducted using MEGA7 (Kumar et al., 2016).

## References

[CIT0001] Kumar S, Stecher G, Tamura K. 2016 MEGA7: molecular evolutionary genetics analysis version 7.0 for bigger datasets. Mol Biol Evol. 33:1870–1874. 27004904 10.1093/molbev/msw054 PMC8210823

[CIT0002] Landvik S, Shailer NFJ, Eriksson OE. 1996 SSU rDNA sequence support for a close relationship between the Elaphomycetales and the Eurotiales and Onygenales. Mycoscience. 37:237–241.

[CIT0003] Mains EB. 1957 Species of tolypocladium parasitic on elaphomyces. Bull Torrey Bot Club. 84:243–251.

[CIT0004] Sun X, Ruan R, Lin L, Zhu C, Zhang T, Wang M, Li H, Yu D. 2013 Genomewide investigation into DNA elements and ABC transporters involved in imazalil resistance in Penicillium digitatum. FEMS Microbiol Lett. 348:11–18. 23952944 10.1111/1574-6968.12235

[CIT0005] Zhang YJ, Zhang S, Liu XZ. 2016 The complete mitochondrial genome of the nematode endoparasitic fungus Hirsutella minnesotensis. Mitochondrial DNA Part A. 27:2693–2694. 10.3109/19401736.2015.1046126 26029879

